# The prevalence of latent tuberculosis infection among healthcare workers in Saudi Arabia: a systematic review and meta-analysis

**DOI:** 10.1016/j.ijregi.2026.101000

**Published:** 2026-09-03

**Authors:** Mohammed Aljabr, Fatima N. Boumarah, Yara Mortada, Ghala Alhmidani, Rakan Almutairi, Jory Msood, Noor Alfaraj, Shatha Abohimed, Adel Almaghrbi, Reham Algethami, Refal Alsaedi, Sadeem Al Maqeet, Abdullah Aljorayid

**Affiliations:** 1 College of Medicine, Qassim University, Buraydah, Saudi Arabia; 2 College of Medicine, King Fahad Hospital of the University, Imam Abdulrahman Bin Faisal University, Dammam, Saudi Arabia; 3 College of Medicine, King Abdulaziz University, Jeddah, Saudi Arabia; 4 College of Medicine, Imam Muhammad Ibn Saud Islamic University, Riyadh, Saudi Arabia; 5 College of Medicine, University of Jeddah, Jeddah, Saudi Arabia; 6 College of Medicine, Najran University, Najran, Saudi Arabia; 7 College of Medicine, King Abdulaziz University Rabigh Branch, Rabigh, Saudi Arabia; 8 Department of Epidemiology, College of Health and Rehabilitation Sciences, Princess Nourah Bint Abdulrahman University, Riyadh, Saudi Arabia; 9 College of Medicine, King Faisal University, Al-Ahsa, Saudi Arabia; 10 Internal Medicine, King Faisal Medical Complex, Taif, Saudi Arabia; 11 Department of Public Health, King Faisal University, Alahsa, Eastern Province, Saudi Arabia; 12 Department of Medicine, College of Medicine, Qassim University, Buraydah, Saudi Arabia

**Keywords:** Latent tuberculosis infection, Healthcare workers, Prevalence, Saudi Arabia, Occupational exposure

## Abstract

**Objectives:**

This systematic review and meta-analysis aimed to estimate the prevalence of latent tuberculosis infection (LTBI) among healthcare workers (HCWs) in Saudi Arabia and identify key associated risk factors.

**Methods:**

A systematic search of PubMed, Web of Science, and MEDLINE and Embase (via Ovid) was conducted for studies reporting LTBI prevalence among HCWs in Saudi Arabia. Data were pooled using a random-effects model. Subgroup analyses assessed variations by diagnostic method, professional category, and nationality.

**Results:**

The overall pooled prevalence was 24% (95% confidence interval [CI] = 16-34%), with high heterogeneity across studies (*I*² = 98.8%). Prevalence was numerically higher in tuberculin skin test (TST)-based studies (27%) than in interferon-gamma release assay (IGRA)-based studies (20%), although this difference did not reach statistical significance (*P* = 0.13). TST-positive/IGRA-negative discordance was the predominant pattern. Expatriate HCWs from high-burden countries showed prevalence rates of 45-46%, compared to 13% among Saudi nationals. Allied health professionals and nurses exhibited higher prevalence trends, though these did not reach statistical significance.

**Conclusion:**

The LTBI burden among HCWs in Saudi Arabia is substantial and largely imported by expatriate workers from high-burden countries, with a possible secondary occupational contribution. TST–IGRA discordance suggests that TST may overestimate prevalence in this bacille Calmette-Guérin-vaccinated population. Based on discordance patterns and supporting evidence, transitioning to IGRA-based screening and implementing targeted surveillance for high-risk groups should be considered.

## Highlights

• This is the first meta-analysis of latent tuberculosis in Saudi healthcare workers.
• Pooled latent tuberculosis prevalence among healthcare workers was 24%
• Expatriate workers from endemic countries had triple the prevalence of nationals.
• Test discordance suggests skin testing may overestimate in bacille Calmette-Guérin-vaccinated groups.
• Findings support transitioning to blood-based screening for this population.

## Introduction

Tuberculosis (TB), caused by *Mycobacterium tuberculosis*, remains a global health challenge. Although it is a preventable and generally curable disease, it persists as the leading cause of death from a single infectious agent. The World Health Organization reported 10.8 million new cases and 1.25 million deaths that occurred worldwide during 2023 [1]. The disease burden exists mainly in low- and middle-income countries, most likely because of the socioeconomic constraints and limited healthcare access, which enable the disease to continue spreading [2].

The reservoir of latent tuberculosis infection (LTBI) is a key part of the TB epidemic. The immune system activates against *M. tuberculosis* antigens during LTBI, but the body does not display any symptoms of active TB [3]. People with LTBI are not contagious, but they remain at risk for disease reactivation, which is estimated at between 5% and 10%, with the highest risk in the first 2 years after infection [1,[3], [4], [5]]. Research shows that LTBI exists in 25% of the human population worldwide [6]. With the reactivation risk, this will continue to cause new active cases, which will prevent the world from reaching its TB elimination goals [1,7].

Healthcare workers (HCWs) are at risk for both LTBI and active TB disease. This risk is driven by the frequency and duration of exposure to infectious patients, the handling of contaminated specimens, and the effectiveness of infection control measures [8,9]. Compared with the general population, HCWs face a twofold increased risk of LTBI and a threefold increased risk of active TB, as shown in a recent systematic review by Uden et al.*,* [10]. Additionally, this occupational risk persists even for HCWs practicing in countries with low TB incidence [11].

Saudi Arabia faces a unique epidemiological situation in regard to TB control. While it is considered a low-burden country with an incidence of 8.4 per 100,000 population [12], the kingdom has a big expatriate workforce originating largely from high-TB-burden regions [13]. Additionally, the annual Hajj and Umrah pilgrimages bring millions of people in close proximity, which makes it easier for airborne diseases to spread [[13], [14], [15]]. This dynamic environment exposes HCWs to a global TB risk profile despite the low local incidence.

The assessment of LTBI prevalence among HCWs in Saudi Arabia is important given these unique demographic and religious factors. This will impact workplace safety and infection control guidance. To our knowledge, no systematic review reports the data on this specific population. This study aimed to systematically review the literature and estimate the pooled prevalence of LTBI among HCWs in Saudi Arabia and identify associated risk factors, with the goal of informing future occupational health strategies.

## Methods

### Study design and protocol

The study was conducted in accordance with the Preferred Reporting Items for Systematic Reviews and Meta-Analyses (PRISMA) guidelines [16]. The study protocol was prospectively registered with PROSPERO (Registration No. CRD420251077993). As this study combined previously published aggregate data, institutional review board approval was not required.

### Search strategy

A comprehensive literature search was executed in December 2025 across the following electronic databases: PubMed, Web of Science, and MEDLINE and Embase (via Ovid). The authors conducted manual screening of reference lists from included articles and relevant reviews to ensure comprehensive coverage. The research question received its structure from the Condition, Context, and Population (CoCoPop) framework [17], which helps develop a focused and rigorous search strategy for prevalence studies. The research focused on LTBI, which affects healthcare facilities throughout Saudi Arabia, focusing on HCWs, including all clinical and nonclinical staff exposed to the hospital environment.

The search strategy used the following keywords and Medical Subject Headings (MeSH) terms: (Latent tuberculosis OR LTBI), (healthcare workers OR occupational exposure), (Tuberculin skin test), and (Prevalence OR Risk factors). Additionally, the search included the following combination: ("epidemiology" OR "prevalence" OR "incidence") AND ("tuberculosis" OR "TB" OR "*Mycobacterium tuberculosis*" OR "*M. tuberculosis*") AND ("Saudi Arabia" OR "Saudi" OR "KSA" OR "Kingdom of Saudi Arabia"). In addition, Google Scholar was used as a supplementary source to identify additional relevant studies through citation tracking and gray literature; it was not treated as a primary database due to its limited reproducibility and inability to execute complex Boolean search strings.

### Eligibility criteria

Studies were selected based on the following inclusion criteria: (1) published in English; (2) conducted in Saudi Arabia; (3) study population consisting of HCWs; (4) diagnosis of LTBI confirmed via tuberculin skin test (TST) or interferon-gamma release assays (IGRAs); (5) reported prevalence data or sufficient raw data to calculate it; and (6) used an observational study design.

Exclusion criteria were: (1) studies conducted outside Saudi Arabia; (2) case reports, reviews, editorials, and conference abstracts without full text; (3) studies focusing solely on active TB; (4) studies where HCW data could not be disaggregated from patient or general population data; and (5) duplicate publications.

### Study selection and data extraction

Two independent reviewers screened titles and abstracts using the Rayyan intelligent systematic review tool [18]. Potentially relevant articles underwent full-text review. Disagreements regarding eligibility were resolved through consensus or consultation with a third senior reviewer.

Data were extracted into a pre-piloted standardized form containing: study characteristics (author, year, region, facility type), participant demographics (sample size, age, sex, nationality, job category), diagnostic methodology (TST cutoff, specific IGRA used), and outcomes (prevalence of LTBI, risk factors).

### Quality assessment

The methodological quality of included cross-sectional studies was evaluated using the Appraisal tool for Cross-Sectional Studies (AXIS) [19]. This tool evaluates study design, sampling strategy, measurement validity, statistical analysis, and reporting clarity. Two reviewers independently scored each study, resolving discrepancies through discussion.

### Meta-analysis

A meta-analysis of proportions was conducted to estimate the pooled prevalence of LTBI using a random-effects model to account for heterogeneity [20]. The logit transformation method was used to stabilize variances, and heterogeneity was assessed using Cochran Q test, τ², and I² statistics (with I² >75% indicating substantial heterogeneity) [21].

Additionally, we conducted a subgroup analysis based on diagnostic tests (TST vs IGRA) and professional groups. Results were presented in multiple forest plots. Subgroup differences were tested using the chi-square test for heterogeneity. We conducted a sensitivity analysis using a leave-one-out method which involved removing one study at a time to determine how each study affected the combined results. The study evaluated publication bias through contour-enhanced funnel plots together with statistical methods which checked for asymmetry in the data. Given the observational nature of included studies, funnel plot findings were interpreted cautiously.

All analyses were conducted in R software (R Foundation for Statistical Computing, Vienna, Austria) using the meta package [22]. The significance level was set at a two-sided *P*-value of <0.05.

## Results

### Study selection

The initial database search yielded a total of 1559 records: 613 from PubMed, 786 from Web of Science, and 160 from Ovid. No additional records were identified through supplementary Google Scholar citation tracking (Figure 1). After removing duplicates, 903 unique records remained for screening. A thorough review of titles and abstracts resulted in the exclusion of 888 records that did not meet the eligibility criteria. Subsequently, the full texts of the remaining 15 articles were assessed for eligibility. Five articles were excluded, leaving 10 studies that met all inclusion criteria. These 10 studies were included in the systematic review and meta-analysis [[23], [24], [25], [26], [27], [28], [29], [30], [31], [32]].

**Figure 1:**
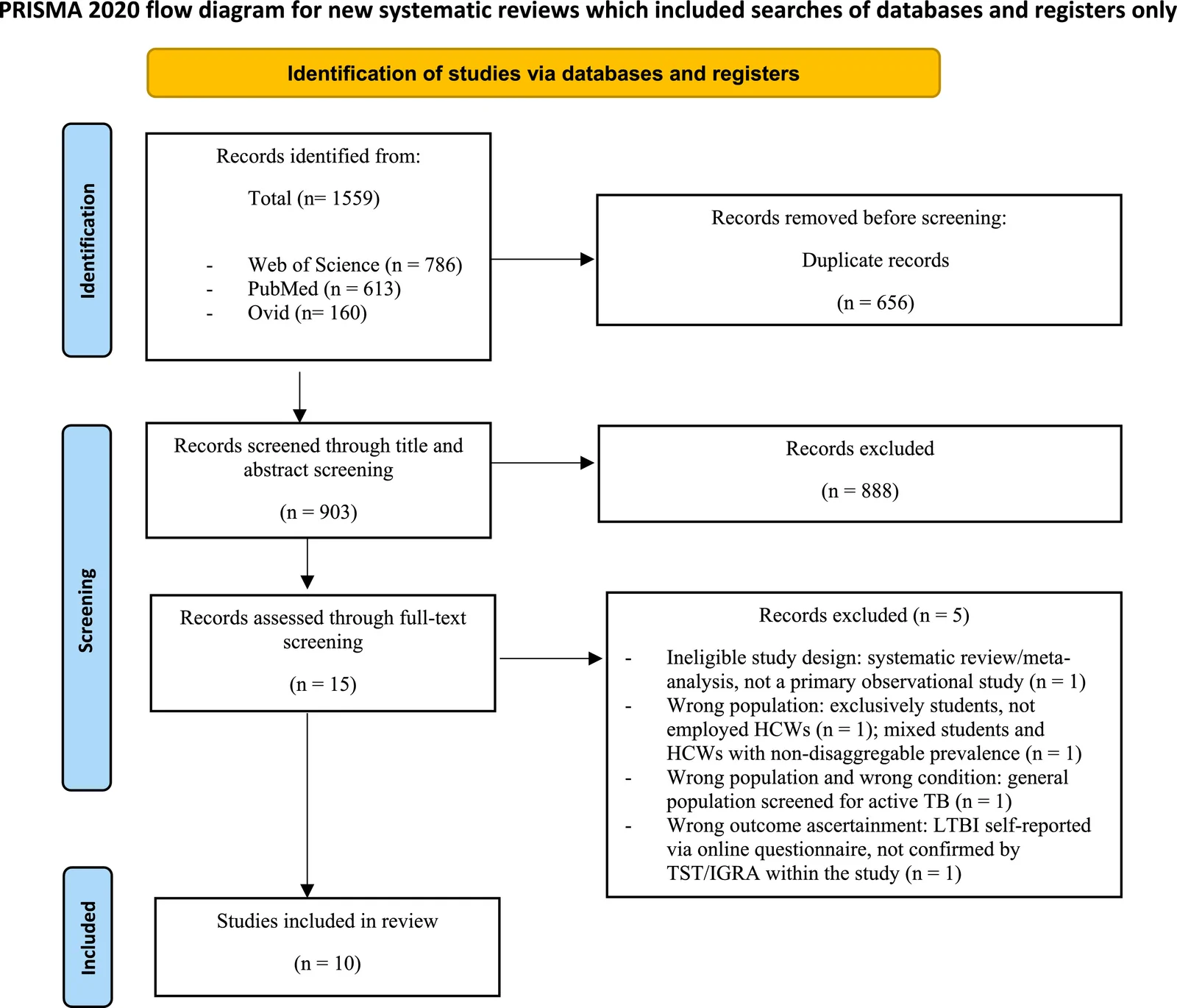
PRISMA flow diagram of study selection process. Abbreviations: HCWs, healthcare workers; IGRA, interferon-gamma release assay; LTBI, latent tuberculosis infection; PRISMA, Preferred Reporting Items for Systematic Reviews and Meta-Analyses; TB, tuberculosis; TST, tuberculin skin test.

### Characteristics of included studies

The 10 included cross-sectional studies were conducted in Saudi Arabia between 2003 and 2024, with sample sizes ranging from 134-3024 participants. Participant ages ranged from 18-60+ years, and the proportion of female participants varied between 33% and 88%.

Nurses were the most frequently represented profession, followed by physicians, allied health staff, laboratory technicians, and nonclinical employees. Some studies also included pharmacists, housekeepers, radiology technicians, and medical students. The participants were of diverse nationalities, with expatriates from high-TB-endemic regions, such as the Philippines and India, often outnumbering Saudi nationals. The studies were conducted in tertiary and secondary care hospitals, as well as general hospitals, reflecting a range of healthcare environments. The different studies reported LTBI prevalence rates which spanned from 8.5%-78.9%. A detailed summary of the characteristics of each study is presented in Table 1.

**Table 1:** Overview of selected studies on LTBI among HCWs.

Study	Study Setting	Participants	Age Range (years)	Sex Distribution	Nationality	Job Title/ Department	Diagnostic Method	Prevelance
Koshak and Tawfeeq, 2003 [23]	Tertiary Academic Hospital	298	21-59	Male: 58 Female: 240	Saudi Arabia: 40 Philippine: 150 India: 36 Pakistan: 31 Nigeria: 24 Other: 17	Laboratories: 60 Neonatal intensive care unit: 26 Paediatric medical ward: 24 Other Departments: 188	TST	235 (78.9%)
Abbas et al., 2010 [24]	Four Tertiary Hospitals	2650	10-50^+^	Male: 1180 Female: 1470	Saudi Arabia: 1254 Middle East: 285 East Asia: 981 Sub-Saharan: 36 Western: 91	Nurse: 1164 Physician: 429 Technician: 423 Medical student: 181 Administrative employee: 453	TST	291 (11%)
Hassan and Diab, 2014 [25]	University Hospital	134	21-60	Male: 72 Female: 62	Saudi Arabia: 97 Middle East: 3 East Asia: 34	Laboratory: 134	IGRA	26 (19.4%)
El-Helaly et al., 2014 *[*26]	Tertiary Care Hospital	1412	19-60	Male: 433 Female: 939	Saudi Arabia: 563 Asia: 686 Africa: 53 Europe: 70 North America: 30 Australia: 10	Nurse: 547 Physician: 197 Other clinical employee: 330 Non clinical employee: 338	TST and IGRA	TST: 421 (29.8%) IGRA: 333 (23.6%)
Hibah and Hasan, 2015 [27]	General Hospital	208	20-50^+^	Male: 91 Female: 117	Saudi Arabia: 24 Middle East: 42 Yemen: 18 Philippine: 83 South Asia: 41	Nurse: 73 Physician: 36 Technician: 29 Administrative employee: 39 Others: 31	TST	47 (22.5%)
Alhajoj et al., 2016 *[*28]	Tertiary Care Hospital	1595	19-60^+^	Male: 512 Female: 1083	Saudi Arabia: 271 Western-Pacific: 694 Eastern Mediterranean: 303 South East Asian: 150 Others: 177	Nurse: 849 Physician: 44 Allied Health: 248 Supportive Services: 348 Administration: 62 Researchers: 44	TST and IGRA	TST: 503 (31.5%) IGRA: 399 (25%)
Bukhary et al., 2018 [29]	Multiple Secondary Care Hospitals	520	22-62	Male: 348 Female: 172	Saudi Arabia: 221 Non-Saudi: 299	Nurse: 160 Physician: 100 Pharmacist: 70 Laboratory Technician: 50 Radiology Technicians: 70 Housekeeper: 70	TST and IGRA	TST: 44 (8.5%) IGRA: 56 (10.8%)
Almohaya et al., 2020 [30]	Tertiary Academic Hospital	3024	20-50^+^	Male: 875 Female: 2149	Saudi Arabia: 750 Philippine: 1341 India: 722 Other: 211	Nurse: 1745 Physician: 123 Allied Health: 318 Laboratory Technician: 49 Radiology Technicians: 43 Non clinical employee: 746	IGRA	733 (24.2%)
Alshahrani et al., 2023 [31]	General Hospital	561	23-71	Male: 150 Female: 411	Saudi Arabia: 51 Non-Saudi: 510	Nurse: 367 Physician: 165 Others: 29	TST	66 (11.8%)
Aldhawyan et al., 2024 [32]	Tertiary Care Hospital	771	18-59	Male: 92 Female: 679	Saudi Arabia: 99 Philippine: 511 Indian: 88 Other: 73	Nurse: 771	TST	266 (34.5%)

Abbreviations: HCWs, healthcare workers; IGRA, interferon-gamma release assay; LTBI, latent tuberculosis infection; TST, tuberculin skin test.

### Diagnostic methods and discordance

Five studies used TST alone, two used IGRA alone, and three utilized both methods. Among the three studies that employed both diagnostic methods, concordant and discordant results were reported.

Across all three studies, concordance between TST and IGRA results varied. El-Helaly et al., reported an overall agreement of 73.7% of results, with most concordance coming from negative results (60.1%) [26]. Similarly, Al Hajoj et al., found 57.7% negative concordance and 14.23% positive concordance [28]. In comparison, Bukhary et al., observed 89.5% negative concordance and 13.6% positive concordance [29].

Discordance between diagnostic methods was a prominent finding in all three studies. The most frequent pattern was TST-positive/IGRA-negative discordance. Among participants who tested positive by TST, a subsequent negative IGRA result was found in 16.3% of the cohort in El-Helaly et al.*,* 54.8% in Al Hajoj et al., and 86.4% in Bukhary et al. Conversely, the TST-negative/IGRA-positive pattern was less common, occurring in 10.1% of participants in El-Helaly et al.*,* 15.7% in Al Hajoj et al., and 10.5% in Bukhary et al., [26,28,29]

### Prevalence of LTBI

The overall pooled prevalence of LTBI across the 10 studies was 24% (95% confidence interval [CI] = 16-34%), calculated using a random-effects model (Figure 2). However, there was a large degree of heterogeneity (*I*² = 98.8%; τ² = 0.0406; *P* <0.0001) among the included studies.

**Figure 2:**
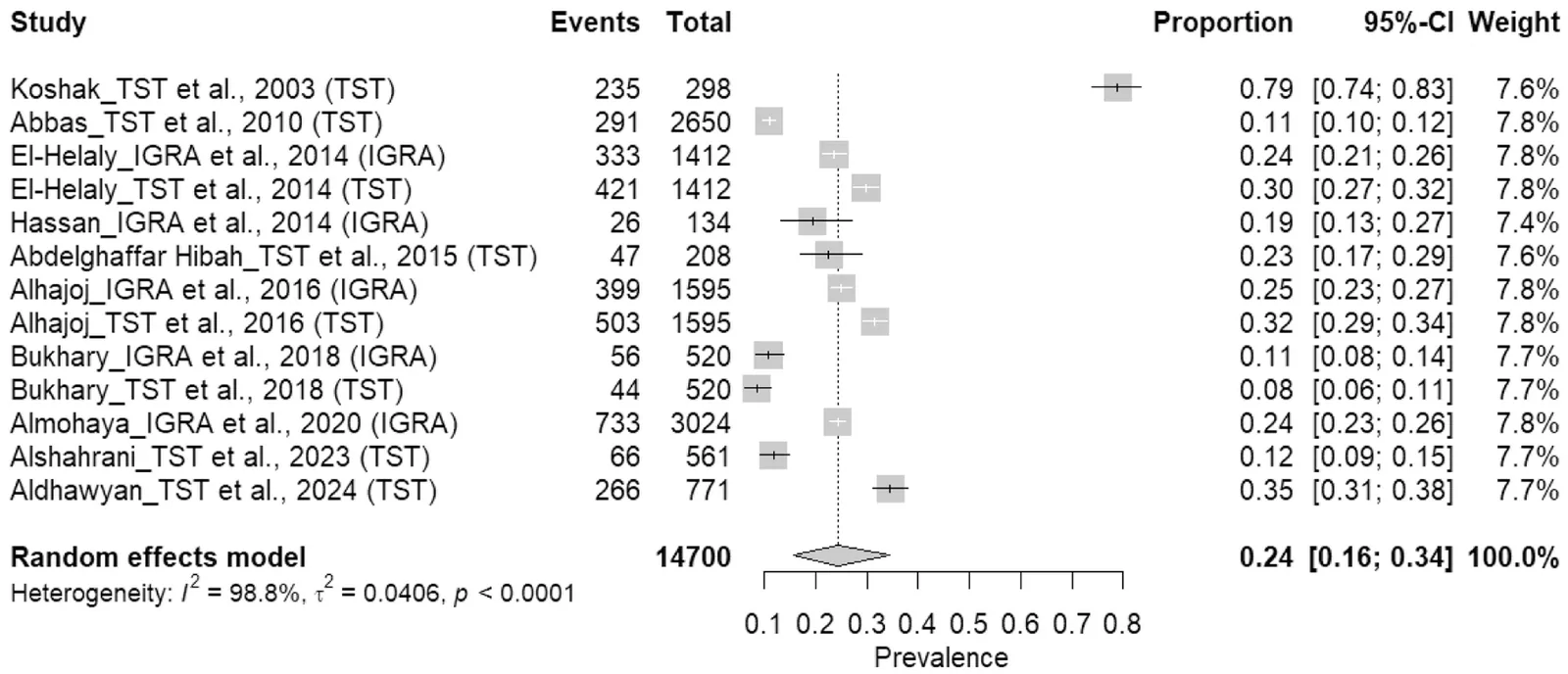
Overall pooled prevalence of LTBI among HCWs. Abbreviations: CI, confidence interval; HCWs, healthcare workers; IGRA, interferon-gamma release assay; LTBI, latent tuberculosis infection; TST, tuberculin skin test.

The subgroup analysis by diagnostic test is shown in Figure 3. The TST-based studies found a pooled prevalence of LTBI of 27% (95% CI = 13-44%), showing high heterogeneity (*I*² = 99.3%, τ² = 0.0640, *P* <0.0001). On the other hand, the IGRA-based studies yielded a pooled prevalence of LTBI of 20% (95% CI = 15-26%), again showing high levels of heterogeneity (*I*² = 93.7%, τ² = 0.0058, *P* <0.0001). No statistically significant difference was found between the prevalence estimates calculated using TST and IGRA (χ² = 2.33, degrees of freedom (df) = 1, *P* = 0.13). The total prevalence estimate pooling the two tests remains at 24% (95% CI = 16-34%).

**Figure 3:**
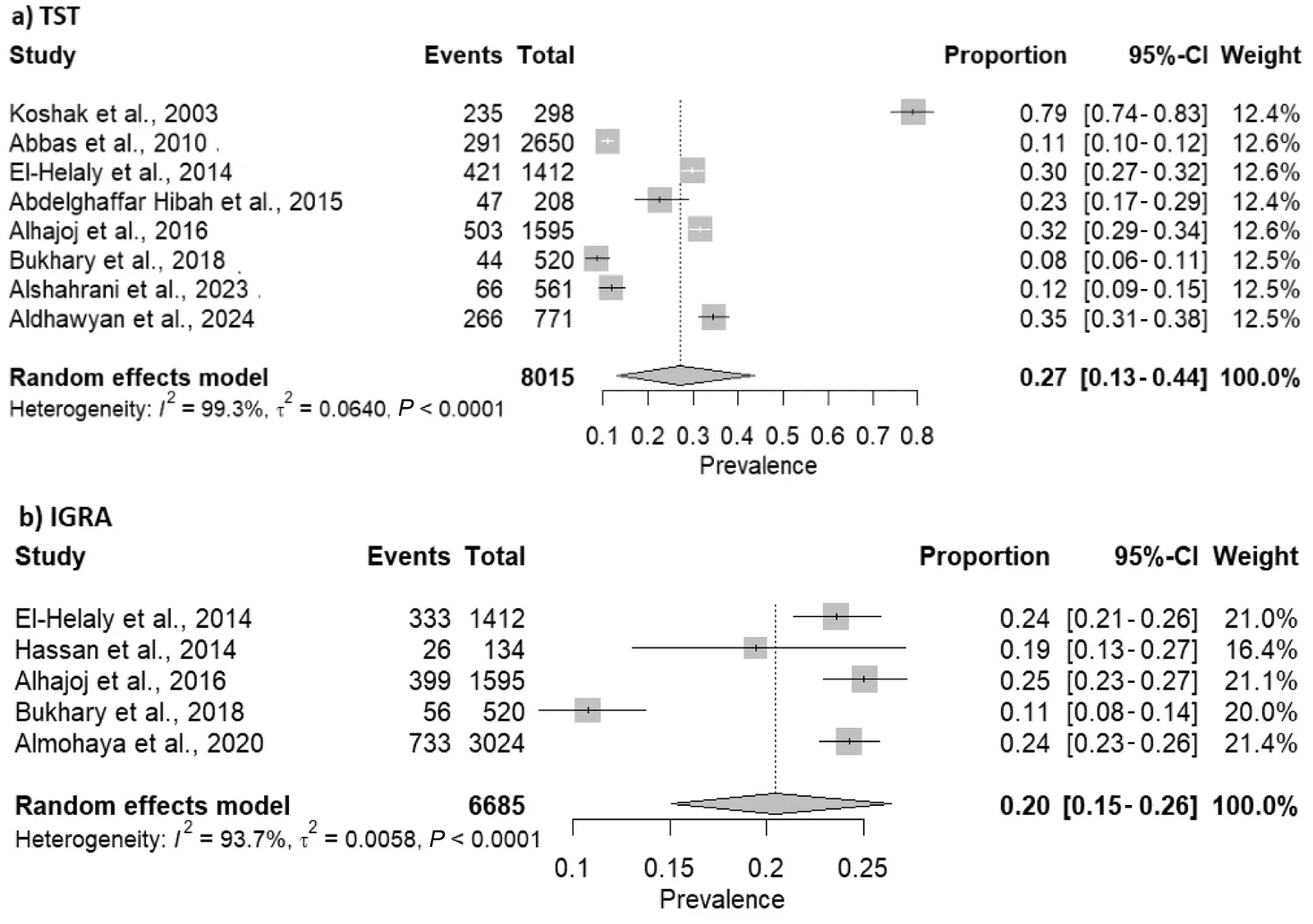
Pooled prevalence of LTBI stratified by diagnostic method. Abbreviations: CI, confidence interval; IGRA, interferon-gamma release assay; LTBI, latent tuberculosis infection; TST, tuberculin skin test.

The prevalence of LTBI varied across healthcare professional categories (Figure 4). Among nurses, the prevalence was 21% (95% CI = 14-29%), with significant heterogeneity (*I*² = 97.4%, τ² = 0.0176, *P* <0.0001). Physicians had a prevalence of 18% (95% CI = 15-22%), with moderate heterogeneity (*I*² = 33.5%, τ² = 0.0015, *P* = 0.17). Allied health professionals showed a prevalence of 24% (95% CI = 16-33%), while pharmacists had a prevalence of 6% (95% CI = 2-14%). Medical students had the smallest LTBI prevalence of 2% (95% CI = 1-6%). Among nonclinical employees, the prevalence was 13% (95% CI = 11-16%). The differences between professional categories were not statistically significant (χ² = 2.33, df = 2, *P* = 0.31).

**Figure 4:**
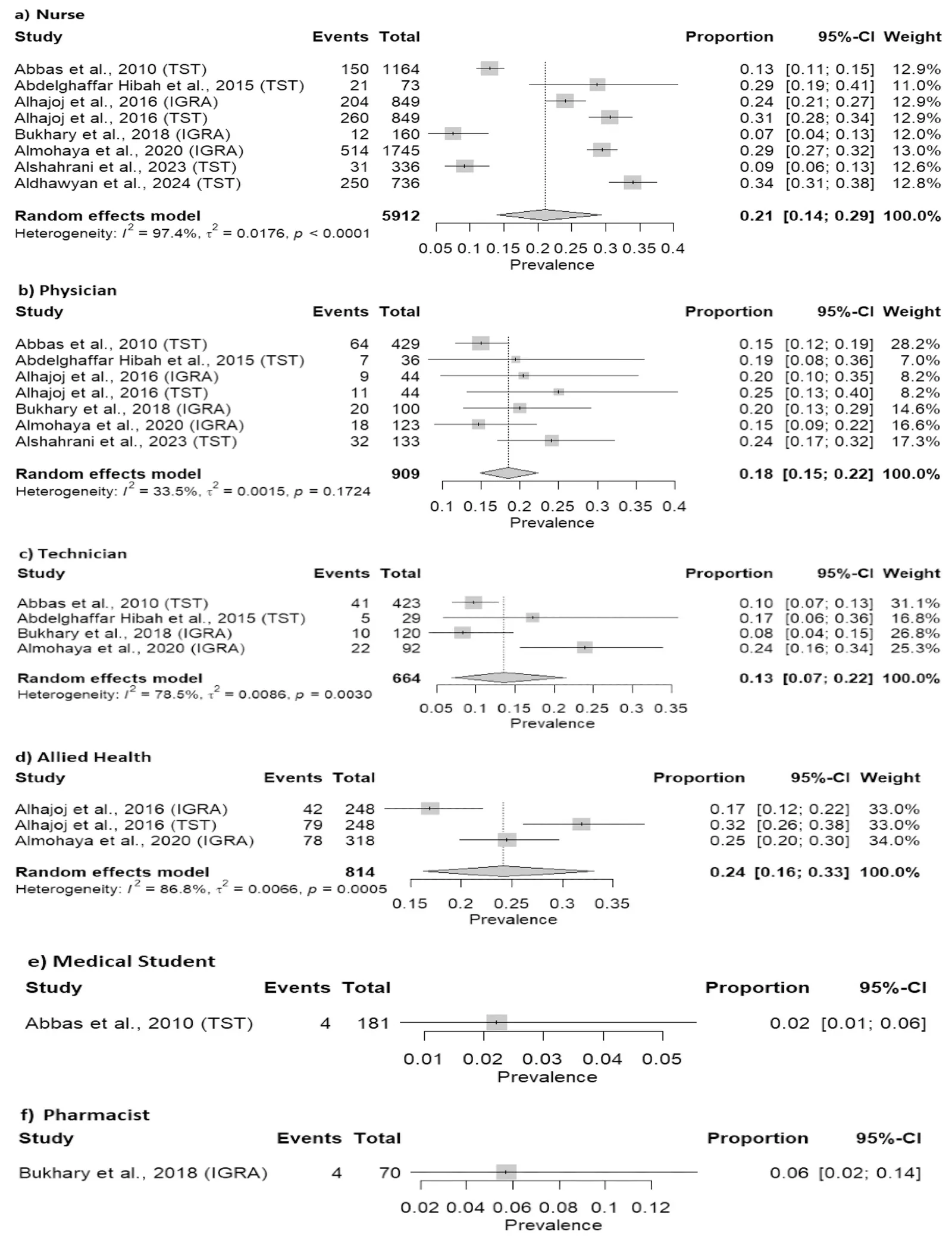
Pooled prevalence of LTBI stratified by professional category. Abbreviations: CI, confidence interval; IGRA, interferon-gamma release assay; LTBI, latent tuberculosis infection; TST, tuberculin skin test.

The analysis of LTBI prevalence stratified by nationality revealed significant variations. Among Saudi HCWs, the prevalence was 13% (95% CI = 4-24%), with significant heterogeneity (*I*² = 94.4%). In comparison, non-Saudi HCWs from endemic countries demonstrated substantially higher prevalence rates. For instance, Indian HCWs had a pooled prevalence of 45% (95% CI = 18-73%), while those from the Philippines had a prevalence of 46% (95% CI = 21-72%), both characterized by high heterogeneity (*I*² > 93%) (Figure S1).

### Assessment of publication bias

A visual inspection of the funnel plot showed asymmetry (Figure 5). The high level of heterogeneity in our research results together with the observational design of prevalence studies makes it more likely that the asymmetry revealed in the funnel plot could be due to inter-study variability rather than publication bias.

**Figure 5:**
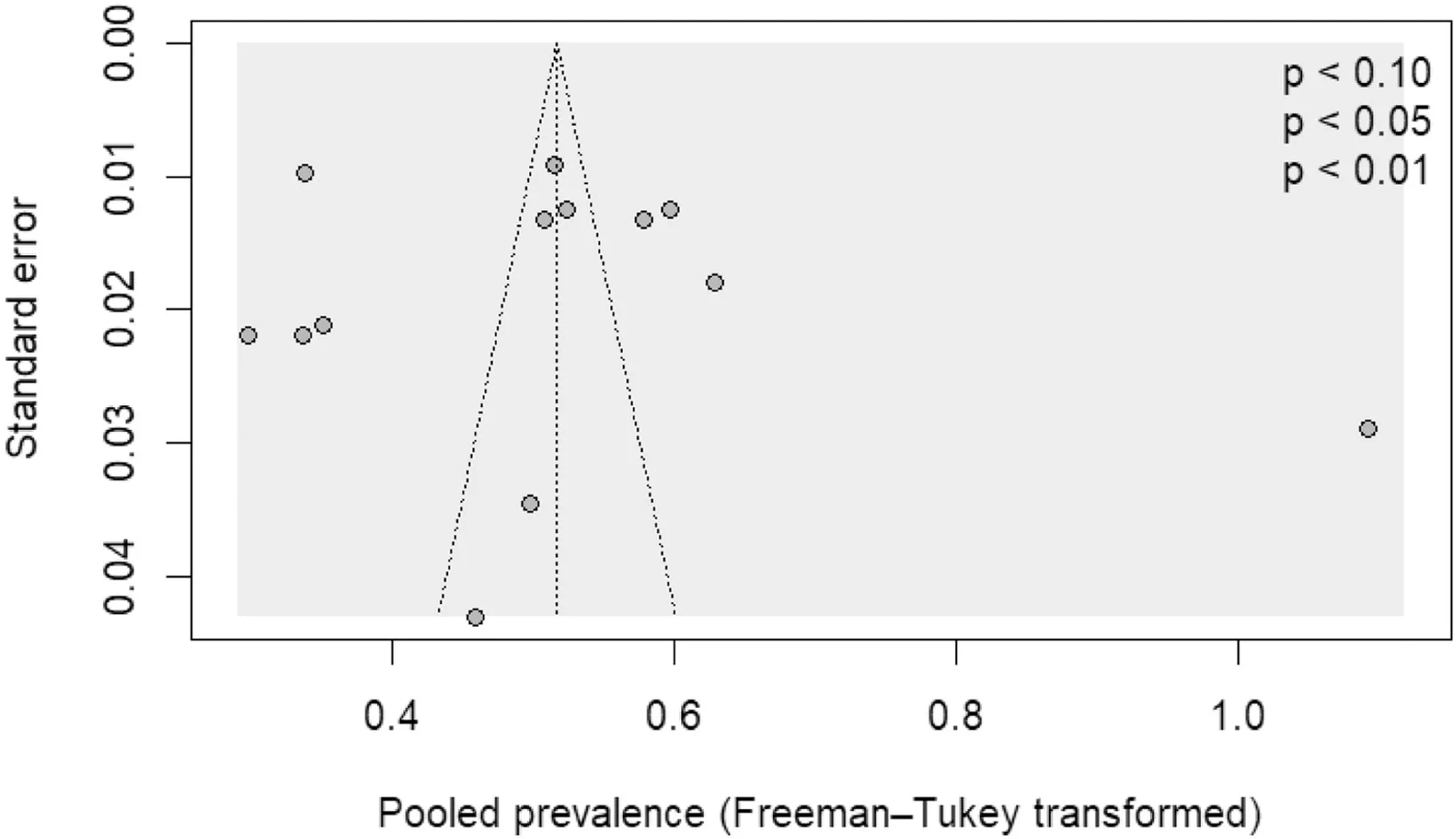
Funnel plot for assessment of publication bias.

### Sensitivity analysis

A leave-one-out sensitivity analysis showed that the pooled prevalence remained stable between 23% and 25%, even when individual studies were excluded. Heterogeneity remained high across all iterations (*I*² ≈ 98-99%), indicating that no single study significantly influenced the overall prevalence estimate (Figure S2).

### Quality assessment and risk of bias

The methodological quality of the included studies was assessed using the AXIS appraisal tool, with scores ranging from 15-17 out of a possible 20. Two studies, Alshahrani et al., and Almohaya et al., received the highest score of 17, reflecting strong methodological quality and a lower risk of bias. In contrast, two studies, Abbas et al., and Al Hajoj et al., scored 15, indicating a moderate risk of bias. A common limitation identified across all studies was the lack of justification for sample sizes (Q7 on the AXIS tool), where all 10 studies scored "No." Despite these limitations, the overall quality of the studies was deemed moderate to high, allowing for confident interpretation of the results, while acknowledging potential biases related to these limitations.

## Discussion

This review provides the first comprehensive assessment of LTBI prevalence among HCWs in Saudi Arabia. The analysis found a pooled LTBI prevalence of 24% (95% CI = 16-34%), which is suggestive of an elevated occupational risk among HCWs. However, this estimate should be interpreted with caution given the extremely high statistical heterogeneity (*I*² = 98.8%), the wide range of primary prevalence rates (8.5-78.9%), and the fact that none of the included studies provided adequate sample size justifications. Accordingly, this pooled figure should be viewed as an approximate summary measure indicating a substantial LTBI burden rather than a precise point estimate. Expatriate HCWs from high-TB-endemic countries were found to have prevalence rates nearly triple those of Saudi nationals, suggesting that the Saudi healthcare sector LTBI burden depends mainly on the composition of its workforce.

The study revealed numerically different levels of LTBI prevalence based on the diagnostic method used. The prevalence rate using TST was 27% compared to 20% using IGRAs, although this difference was not statistically significant (*P* = 0.13). Similarly, the professional categories showed no statistically significant difference (*P* = 0.31), though point estimates suggested that nurses and allied health professionals may have higher prevalence than other groups. These nonsignificant trends warrant further investigation in adequately powered primary studies. The findings illustrate the complexity of LTBI screening in a multinational workforce and underscore the importance of developing context-specific infection control policies.

The observed pooled prevalence of 24% among HCWs appears higher than the 9.8% prevalence reported in the general Saudi population [33]. Even when isolating Saudi national HCWs, our study found a pooled prevalence of 13%, which appears elevated compared to the general public. However, it should be noted that no formal statistical comparison was performed between these estimates, as they are derived from separate meta-analyses with different populations and methodologies. Nevertheless, the observed difference is suggestive of a possible occupational contribution to LTBI risk. Despite this limitation, when viewed through a global lens, the prevalence among HCWs in Saudi Arabia is comparatively moderate. Research studies of HCWs in low- and middle-income countries have reported prevalence rates as high as 49% [34], with similar rates (47%) observed in studies focusing on high-TB-burden nations [35]. The lower prevalence in Saudi Arabia reflects the country's declining TB incidence, which dropped from 10.55 per 100,000 in 2015-8.76 per 100,000 in 2019 [36]. The country achieved this relative success through strong infection control measures and implemented strict pre-employment screening policies for all workers.

The review shows that different national groups have different infection rates, which makes it difficult to compare against other countries that have low-incidence rates. Our analysis showed that HCWs from India and the Philippines had infection rates of 45% and 46%, respectively, which matched the rates in their home nations instead of the Saudi population average. This suggests that a significant portion of the HCWs’ LTBI in Saudi hospitals is imported rather than locally acquired. This finding aligns with other research which shows that TB epidemiological patterns among HCWs often follow the epidemiology of their foreign workforce instead of the local population [9,11,37].

The selection of diagnostic tests raised concerns about the specificity of the screening protocol, particularly for the bacille Calmette-Guérin (BCG)-vaccinated population. The TST-based studies reported a 27% pooled prevalence compared to 20% found in IGRA-based studies, although this difference did not reach statistical significance in the subgroup analysis. Similar findings were shown in another study, where they reported a 10% absolute difference between the TST and IGRA estimates [34]. The most prominent pattern of this difference was TST-positive/IGRA-negative. This is most likely attributed to the previous BCG vaccine and nontuberculous mycobacteria infection. In a review article by Nienhaus et al.*,* they reported that BCG vaccination and migration background status together accounted for 85% of cases of this pattern [38]. Given that many expatriate HCWs in Saudi Arabia originate from countries with universal BCG vaccination policies, TST likely overestimates the true burden of LTBI in this setting. The occurrence of TST-negative/IGRA-positive discordance represents a rare situation that could lead to a missed diagnosis. This pattern might occur because of cutaneous anergy, immunosuppression, or in older populations. Gao et al.*,* show that IGRA tests are more sensitive, particularly for this particular population, and more specific, which decreases the chances of overdiagnosis [39]. Moreover, while dual-testing strategies (IGRA confirmatory testing for individuals with positive TST results) have been explored, the authors found that the positive predictive value of IGRA alone was comparable to dual-testing strategies [39]. Given the observed discordance patterns and the well-documented limitations of TST in BCG-vaccinated populations, the scientific basis for using TST as the main screening method in a workforce with multiple nationalities and high vaccination rates is questionable, lending support to considering IGRA-based screening protocols.

The analysis of occupational categories revealed that allied health professionals (24%) and nurses (21%) had numerically higher prevalence than pharmacists (6%) and medical students (2%); however, these differences did not reach statistical significance (*P* = 0.31). While external literature has reported that frontline HCWs encounter greater exposure risk [34,35,40], the present analysis was unable to confirm this pattern statistically, likely due to limited subgroup sample sizes and high between-study heterogeneity. Other risk factors reported in the included studies were older age [[24], [25], [26], [27],[29], [30], [31], [32]], longer duration of employment [25,29,32], and working in high-risk areas like intensive care units or emergency departments [29,30,32].

The strengths of this review include its comprehensive search strategy and focus on a specific national context; however, some limitations must be acknowledged. First, a high degree of heterogeneity (*I*² = 98.8%) was observed across the included studies. This is a well-recognized characteristic of prevalence meta-analyses, where estimates are inherently influenced by differences in population demographics, geographic context, healthcare settings, and diagnostic methodology. As recommended by Borges Migliavaca et al., the purpose of pooling in this context is not to produce a single definitive estimate but rather to provide a summary measure while systematically exploring sources of heterogeneity through subgroup and sensitivity analyses, which we have performed [41].Second, the cross-sectional design of the included studies prevents the assessment of LTBI conversion rates or progression to active TB. Third, we were unable to fully adjust for the BCG status of participants in the TST subgroup analysis due to inconsistent reporting in primary studies. Fourth, none of the included studies reported using the two-step TST method, which establishes a true baseline by identifying boosted reactions in serially tested individuals. Without this approach, positive TST results from repeated occupational screening may include boosted responses misclassified as new infections, potentially inflating prevalence estimates, particularly in this heavily BCG-vaccinated population where periodic screening is routine. This may have further inflated TST-based prevalence estimates beyond the effect of BCG cross-reactivity alone.

The observed TST-positive/IGRA-negative discordance patterns, together with external evidence supporting IGRA's superior specificity in BCG-vaccinated populations [38,39], support consideration of transitioning to IGRA tests as the standard screening tool for HCWs. This could help decrease the requirement for unnecessary chest x-rays and chemoprophylaxis treatments for the high rate of individuals with TST-positive/IGRA-negative results, which will offer long-term cost reduction. Given that the majority of the detected LTBI burden appears to be imported, screening protocols should prioritize robust pre-employment baseline testing, particularly for expatriates arriving from high-TB-burden countries. For institutions where IGRA cannot be implemented, protocols should be risk-stratified, directing resources toward high-risk populations and those working in high-risk areas. Additionally, the presence of LTBI even among Saudi nationals (13%) suggests that some degree of local occupational transmission may be occurring, reminding us of the importance of continuing infection control measures, including rigorous fit-testing of N95 respirators and continuous education regarding the early recognition of TB symptoms.

In conclusion, while Saudi Arabia is a low-incidence country, HCWs appear to have a higher LTBI prevalence than the general population, although this comparison is indirect and was not formally tested for statistical significance. The evidence indicates that most of this burden is imported, with expatriate HCWs from high-TB-burden countries demonstrating prevalence rates that closely mirror the epidemiology of their countries of origin. Nevertheless, occupational exposure may also contribute, as suggested by the numerically higher prevalence observed among Saudi national HCWs compared to the reported general population estimate. Addressing this dual challenge requires robust pre-employment and periodic LTBI screening, particularly targeting expatriates from endemic regions, alongside continued strict adherence to infection control measures to minimize local occupational transmission.

## CRediT authorship contribution statement

**Mohammed Aljabr:** Conceptualization, Data curation, Investigation, Methodology, Writing – original draft. **Fatima N. Boumarah:** Data curation, Investigation, Methodology, Writing – original draft. **Yara Mortada:** Data curation, Investigation, Writing – original draft. **Ghala Alhmidani:** Data curation, Investigation, Writing – original draft. **Rakan Almutairi:** Data curation, Investigation, Writing – original draft. **Jory Msood:** Data curation, Investigation, Writing – original draft. **Noor Alfaraj:** Data curation, Investigation, Writing – original draft. **Shatha Abohimed:** Data curation, Investigation, Writing – original draft. **Adel Almaghrbi:** Data curation, Investigation, Writing – original draft. **Reham Algethami:** Data curation, Investigation, Writing – original draft. **Refal Alsaedi:** Data curation, Investigation, Writing – original draft. **Sadeem Al Maqeet:** Data curation, Investigation, Writing – original draft. **Abdullah Aljorayid:** Conceptualization, Methodology, Project administration, Supervision, Validation, Writing – review & editing.

## Declaration of competing interest

The authors have no competing interests to declare.

## Appendix Supplementary materials

Image, application 1
