# Supplementary material for: The prevalence of latent tuberculosis infection among healthcare workers in Saudi Arabia: a systematic review and meta-analysis
Source: IJID Reg. 2026 Sep 3;21:101000. doi: 10.1016/j.ijregi.2026.101000 (PMC13627945; doi:10.1016/j.ijregi.2026.101000)
Supplement: Supplementary file 1 [file mmc1.docx]

**Figure S1: Subgroup analysis of LTBI prevalence by geographic region/ nationality**.

**Figure S2: Leave-one-out sensitivity analysis.**
